# Effects of Blood Flow Restriction Training on the Upper Extremities: A Scoping Review

**DOI:** 10.7759/cureus.79876

**Published:** 2025-03-01

**Authors:** Michael J Sinnott, Nicole Schneider, Pradeep Vanguri

**Affiliations:** 1 Medicine, Florida International University, Herbert Wertheim College of Medicine, Miami, USA; 2 Medicine, Dr. Kiran C. Patel College of Osteopathic Medicine, Nova Southeastern University, Davie, USA; 3 Health and Human Performance, Nova Southeastern University, Fort Lauderdale, USA

**Keywords:** athletes, athletic therapy, blood flow restriction training, elbow injury, rehabilitation, shoulder injury, sports medicine, upper extremities

## Abstract

High-intensity training leads to muscle growth in the upper extremities but at the expense of a greater risk of damaging joints, ligaments, or tendons due to this region being more prone to injury. In contrast, low-intensity resistance training implements a low load with high repetition, which is safer but yields less muscle hypertrophy and greater time consumption. Blood flow restriction (BFR) training potentially provides a solution as it achieves the muscle growth and strengthening of high-intensity resistance training while safely performing low-intensity resistance training. This technique reduces venous return and arterial blood flow to the targeted limb, creating a physiological environment to induce muscle hypertrophy, increase strength, and prolong endurance while minimizing tissue stress. This study will analyze literature within the past 10 years on the usage of BFR training in the upper extremities. An extensive search on PubMed was performed using the term “blood flow restriction training upper extremity,” which resulted in 98 articles that were narrowed down to 17 based on clinical trials and relevance to upper extremities. Results from this study demonstrate that BFR training enhances muscle hypertrophy and strength in the upper extremity muscles, including the biceps brachii and triceps brachii at low loads. Additionally, BFR training has been shown to promote adaptations in non-occluded muscles proximal and contralateral to the cuff location. Clinical applications include postoperative recovery, rehabilitation of rotator cuff injuries, and treatment of tendinopathies. BFR training offers an effective alternative to high-load resistance training for the upper extremities with promising applications in rehabilitation and injury prevention. Future research is necessary to address long-term effects, optimize protocols, and diversify its applications.

## Introduction and background

Blood flow restriction (BFR) training, developed in 1966 by Yoshiaki Sato in Japan, involves combining exercises performed with low-load resistance training along with arterial and venous occlusion achieved using a tourniquet [1,2]. This technique has gained popularity with both athletes and physicians with increased implementation in clinical practice [3,4]. 

Traditionally, high-load exercises have been the gold standard in encouraging strength and muscle gains [5]. Although this is the most popular method, it is not ideal for populations such as elderly populations, athletes recovering from an injury, or individuals with nonfunctioning extremities. BFR training's increased practicality is due to its ability to replicate the results of high-load resistance exercises while minimizing mechanical stress exerted on muscles, tendons, and joints [6,7]. These benefits make BFR training a useful tool to maximize muscle growth when heavy loads are not feasible.

BFR training involves applying a tourniquet cuff to the proximal limb causing a restriction of arterial blood flow into the limb and venous blood flow out of the limb. This creates a more hypoxic state during the exercise, which increases metabolic stress in conjunction with the mechanical tension from the exercise. The metabolic stress causes a cascade of events, including hormone production, fast-twitch fiber recruitment, cellular swelling, and increased reactive oxygen species [8,9]. These factors lead to a cascade of events that ultimately result in increased muscle hypertrophy despite having minimal amounts of mechanical tension [10].

There are unique challenges to strengthening the upper extremity muscles due to factors such as anatomical vulnerability and higher risk of injury in this location, especially during high-intensity exercises. Muscles in this region, such as the rotator cuff and biceps brachii, are typically smaller with a wide range of motion, making them more delicate than the muscles in the lower extremities. Due to these frequent, complex fine motor movements, there is an increased susceptibility to injury. The potential for BFR training is substantial, particularly in locations where direct occlusion is difficult to achieve, such as the rotator cuff muscles. Due to the ability of BFR training to impact the contralateral limb through systemic and neural adaptions, this opens a new avenue for rehabilitation techniques that can be used in clinical practice [6,11]. Its unique cross-training mechanism becomes increasingly important when the target limb is unable to be trained, such as in a stroke or spinal cord injury.

There has been extensive research surrounding BFR training in the lower extremities that has demonstrated its efficacy in improving muscle strength, size, and endurance [12,13]. Studies focused on the upper extremities remain limited due to challenges in anatomical complexity and a relatively smaller number of clinical trials specifically for the upper extremity, making it important to address this gap in the literature. This review aims to address the gap in the literature focusing on BFR training in the upper extremities, specifically pertaining to its mechanism of action, efficacy in strengthening muscles, and potential for clinical applications. A systematic evaluation of peer-reviewed articles published within the last decade was performed to provide insights into the role of BFR training in providing a safer, more effective alternative to traditional high-intensity training for the upper extremities.

## Review

Study design

**Figure 1:**
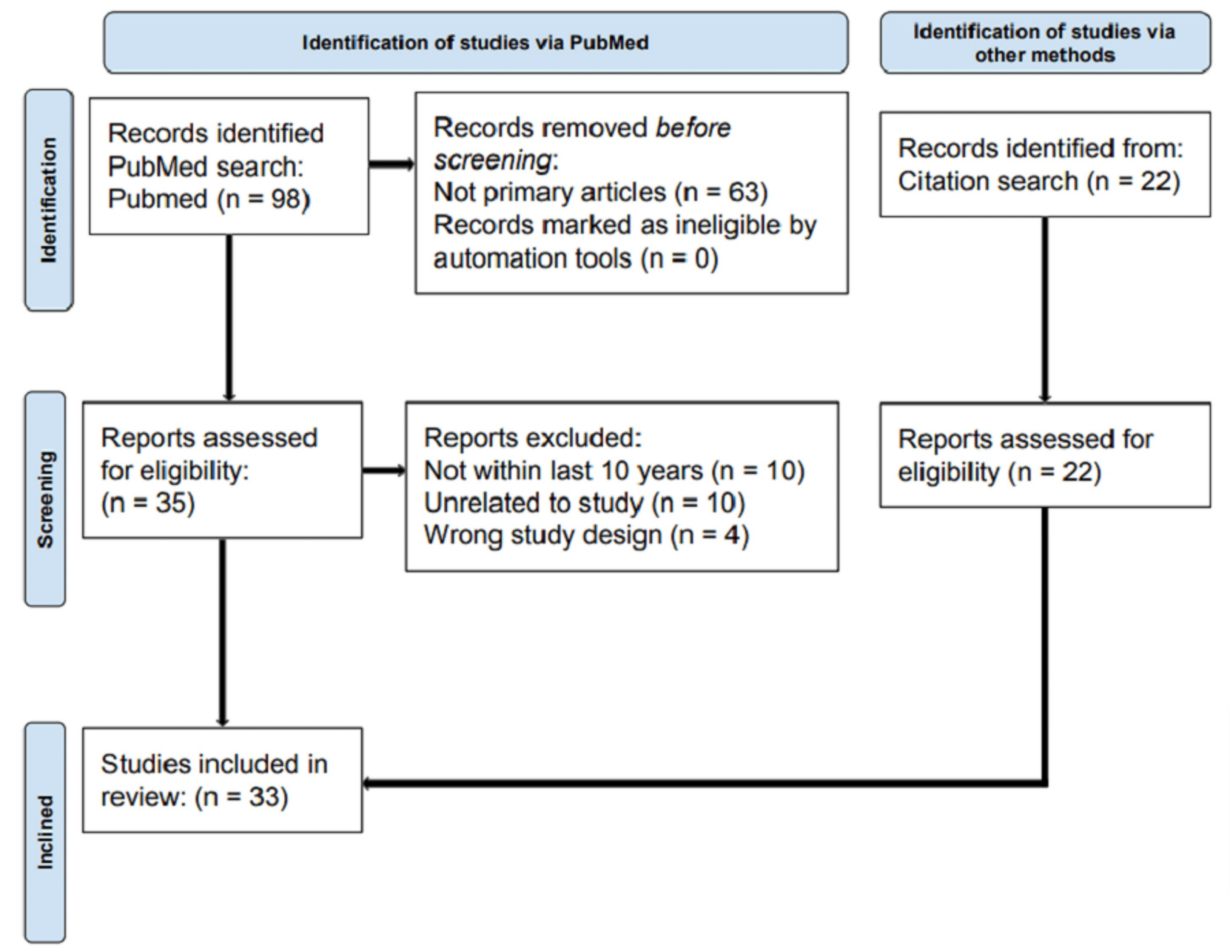
Flow diagram of studies identified via PubMed and other methods

In this literature review, the articles collected and analyzed were previously peer-reviewed published articles on the PubMed database. A comprehensive search on PubMed was performed with the search term “blood flow restriction training upper extremity”, where 98 articles were discovered. The articles were then filtered to include clinical trials and/or randomized controlled trials and exclude any articles that were not primary papers, which yielded 35 articles. An additional filter was placed so that the articles were within the years of 2014 to 2024, reducing the total to 25 articles. The reviewed 25 articles were filtered for wrong study designs, lack of adequate results, or incorrect focus, such as on lower extremities, which produced a total of 11 articles. Citation search was also included when conducting the review, which added 22 more articles and yielded a total of 33 articles. Screening and eligibility of each of the articles for the review were determined utilizing a flow diagram shown in Figure 1.

Challenges of strengthening upper extremity muscles

When strengthening the upper extremities, there are many challenges that must be faced. The shoulder, elbow, and wrist are more vulnerable and prone to injury due to anatomical and physiological characteristics. These locations are composed of smaller, more fragile muscles that are responsible for a wide range of motion and fine motor movements. An example of this is the rotator cuff muscles (supraspinatus, infraspinatus, teres minor, and subscapularis), which are responsible for stabilization of the shoulder. The muscles of the rotator cuff are predisposed to tears due to their small size, extensive range of motion, and support of a less stable glenohumeral joint. In contrast, the lower extremities can withstand greater force due to larger muscles and more sturdy joint structures with thicker cartilage and ligaments.

Because of the feeble nature of the upper extremities, how they are strengthened must be carefully approached. Traditionally, upper extremity strengthening is achieved through high-intensity exercises where there is a high load and low repetition. By using a high load, there is increased tensile stress on the muscle, which promotes muscle growth. This technique is effective in muscle growth, but delicate muscles such as those with the rotator cuff are at an increased risk of injury. In activities such as CrossFit that have a heavy focus on high-intensity training exercises, there was a significant increase in the rate of upper extremity injury in the shoulder, elbow, and wrists when compared to traditional weightlifting exercises [14]. This suggests that high-intensity workouts can have a greater risk of causing injury if not careful.

There are also various other factors that contribute to risk of upper extremity injury, including age and previous injuries. Age has a large impact on occurrence of rotator cuff injury, showing a prevalence of 9.7% in patients 20 years and younger compared to 62% in patients 80 years or older [15]. This can be due to a variety of factors, such as decreased vascularity and weakened muscles, tendons, and ligaments. Strengthening of the upper extremities becomes increasingly concerning when there are preexisting injuries, such as in patients with a torn rotator cuff, as there was a post-operative retear rate of 14% [16].

The upper extremities' anatomical vulnerabilities and sensitive response to exercise and other risk factors make it increasingly important to find a safe yet effective approach to strengthening the upper extremities while minimizing excessive joint and tendon stress. BFR training offers a promising solution by focusing on low-load resistance while yielding results similar to high-load resistance, which can be particularly beneficial for the upper extremities, where high-intensity exercises pose a risk.

Blood flow restriction training: mechanisms and general benefits

BFR training uses a cuff to compress a limb, which temporarily restricts blood flow to a muscle group while exercising. This tricks the body into thinking it is under heavy exertion, which leads to a cascade of events that cause muscle growth. There are two general mechanisms by which BFR training encourages hypertrophic effects. The first is by increasing levels of metabolic stress. When blood flow is restricted to a muscle group, this creates a hypoxic environment and, as a result, leads to the build-up of metabolites that encourage muscle hypertrophy through a variety of mechanisms, including increased hormone production, fast-twitch fiber recruitment, cellular swelling, and increased reactive oxygen species [9]. The buildup of metabolites causes an increase in amino acid transport into cells, therefore promoting the synthesis of contractile proteins such as actin and myosin, leading to muscle hypertrophy [10]. The second mechanism behind BFR training causing muscle growth is due to mechanical tension, which works synergistically with metabolic stress. However, the contribution from mechanical tension in BFR training is believed to be minimal compared to its role in traditional high-load resistance training [9]. Table 1 addresses the key points in the literature that will be discussed throughout this review. 

**Table 1:** Summary points of blood flow restriction training articles BFR: blood flow restriction; UE: upper extremity; BFR-HL: blood flow restriction-high load; BFR-LL: blood flow restriction-low load

Author(s), Year	Title	Summary Points
Nicolay et al., 2022 [1]	Upper extremity injuries in CrossFit athletes—a review of the current literature	Reviews the high rate of CrossFit training injuries in the upper extremities -shoulder, elbow, and wrist compared to traditional weight training, elite gymnastics, and Olympic style lifting.
Codding et al., 2018 [2]	Natural history of degenerative rotator cuff tears	Summarizes the advances in understanding rotator cuff diseases along with the healing and clinic outcomes for small tears in young patients.
Hughes et al., 2017 [3]	Blood flow restriction training in clinical musculoskeletal rehabilitation: a systematic review and meta-analysis	Reviews the effectiveness of low-load BFR training compared to low-load training alone as a clinical rehabilitation tool.
Vanwye et al., 2017 [4]	Blood flow restriction training: implementation into clinical practice	Low-load BFR effectively increases muscle hypertrophy, strength, and functional recovery in the upper extremity.
Garber et al., 2011 [5]	American College of Sports Medicine position stand. Quantity and quality of exercise for developing and maintaining cardiorespiratory, musculoskeletal, and neuromotor fitness in apparently healthy adults: guidance for prescribing exercise	The American College of Sports Medicine recommends adults to exercise for ≥150 min/week with moderate activity or ≥75 min/week with vigorous activity with resistance, flexibility and neuromotor exercises.
Routledge et al., 2023 [6]	Re-tear rates following rotator cuff repair surgery	A common complication post rotator cuff repair surgery is re-tear most likely due to increasing age.
Pearson et al., 2015 [7]	A review on the mechanisms of blood-flow restriction resistance training-induced muscle hypertrophy	Another alternative to muscle growth is the use of BFR training with lower exercise intensities rather than solely resistance training.
Takarada et al., 2000 [8]	Rapid increase in plasma growth hormone after low-intensity resistance exercise with vascular occlusion	Lightweight resistance exercises with occlusion increase the secretion of growth hormone by regional accumulation of metabolites.
Lin et al., 2024 [9]	Differential training benefits and motor unit remodeling in wrist force precision tasks following high and low load blood flow restriction exercises under volume-matched conditions	BFR-HL exercises yielded greater muscle strength, compared to BFR-LL exercises which were found to increase improvement in precision control.
Kara et al., 2024 [10]	Blood flow restriction training in patients with rotator cuff tendinopathy: a randomized, assessor-blinded, controlled trial	The usage of low-load BFR training in rotator cuff tendinopathy allowed for an increase in bicep thickness and shoulder internal rotation strength compared to no BFR; however, no increased improvement was seen in external rotation strength/function between both groups.
Loenneke et al., 2011 [11]	Blood flow restriction: the metabolite/volume threshold theory	In the past, muscle hypertrophy was acquired through increasing load; however, BFR training is another modality which allows for muscle hypertrophy and strength with a lighter load.
Tollefson et al., 2024 [12]	Lower extremity blood flow restriction training in athletes significantly improves strength-related outcomes in 58% of studies compared to non-blood flow restriction control	Lower extremity BFR led to strength gains in 8% of studies in comparison to non-BFR groups showing an increase in muscle size, endurance, and performance.
Ma et al., 2024 [13]	Blood flow restriction combined with resistance training on muscle strength and thickness improvement in young adults: a systematic review, meta-analysis, and meta-regression	BFR with resistance training shows comparable increases in muscle strength and thickness while reducing injury risk with optimal occlusion pressure around 50%.
Bowman et al., 2020 [14]	Upper-extremity blood flow restriction: the proximal, distal, and contralateral effects-a randomized controlled trial	It was seen that low-weight BFR training allowed for a greater increase in strength and hypertrophy in the UEs compared to a control group of no BFR.
Brandner et al., 2015 [15]	Unilateral bicep curl hemodynamics: low-pressure continuous vs high-pressure intermittent blood flow restriction	A decrease in hemodynamic stress was noted in groups with continuous low-pressure BFR training compared to intermittent high-pressure BFR training.
Lambert et al., 2021 [16]	Blood flow restriction training for the shoulder: a case for proximal benefit	Utilizing BFR with low-intensity exercises increased shoulder and arm mass, strength and endurance compared to solely low-intensity exercises of the rotate cuff muscles.
Hill 2020 [17]	Eccentric, but not concentric blood flow restriction resistance training increases muscle strength in the untrained limb	A different modality to increasing muscle function/strength is the usage of low-load eccentric BFR rather than low-load concentric BFR.
Curty et al., 2018 [18]	Blood flow restriction attenuates eccentric exercise-induced muscle damage without perceptual and cardiovascular overload	It was seen that high-intensity eccentric BFR exercise had no increase in cardiovascular response; however, it induced muscle damage.
Laurentino et al., 2016 [19]	The effect of cuff width on muscle adaptations after blood flow restriction training	Low-load BFR exercise with a narrow cuff versus a wide cuff both increased elbow flexion strength and cross-sectional area similarly.
Madarame et al., 2008 [20]	Cross-transfer effects of resistance training with blood flow restriction	Blood flow restriction exercises led to significant hypertrophy and strength gains in both the trained and untrained arms.
Patten et al., 2001 [21]	Adaptations in maximal motor unit discharge rate to strength training in young and older adults	Resistance training caused a 25% increase in max voluntary force in young adults and 33% in older adults.
Shima et al., 2002 [22]	Cross education of muscular strength during unilateral resistance training and detraining	Unilateral resistance training causes a significant increase in maximum voluntary isometric contraction, electromyographic activity, and voluntary activation in trained and contralateral limbs suggesting neural mechanism.
Hill et al., 2021 [23]	Patterns of responses and time-course of changes in muscle size and strength during low-load blood flow restriction resistance training in women	Low-load BFR resistance training yielded an increase in muscle size early on during training prior to a real increase in muscle strength.
Roehl et al., 2023 [24]	Optimal blood flow restriction occlusion pressure for shoulder muscle recruitment with upper extremity exercise	Limb occlusion during BFR leads to increased shoulder muscle activation up to 50% occlusion.
Held et al., 2023 [25]	Low-intensity climbing with blood flow restriction over 5 weeks increases grip and elbow flexor endurance in advanced climbers: A randomized controlled trial	In climbers, BFR increased grip and elbow flexor endurance, but both BFR and non-BFR climbers had similar increases in strength.
Lambert et al., 2023 [26]	Rotator cuff training with upper extremity blood flow restriction produces favorable adaptations in division IA collegiate pitchers: a randomized trial	BFR low-load resistance exercises allowed for an increase in shoulder lean mass and endurance in collegiate baseball pitchers while keeping proper mechanics which could allow for a new modality of strengthening with injury prevention.
Nakajima et al., 2006 [27]	Use and safety of KAATSU training: results of a national survey	KAATSU training, which involves restricted blood flow, has been demonstrated as a safe and healthy alternative for training athletes and individuals with various health conditions.
Anderson et al., 2022 [28]	Overall safety and risks associated with blood flow restriction therapy: a literature review	BFR training is generally safe in rehabilitation and performance uses with adverse events occurring in a small number of individuals, usually with comorbid conditions.
de Queiros et al., 2021 [29]	Application and side effects of blood flow restriction technique: a cross-sectional questionnaire survey of professionals	BFR is commonly used for muscle hypertrophy and rehabilitation with minimal side effects, most commonly tingling and delayed muscle soreness.
Minniti et al., 2020 [30]	The safety of blood flow restriction training as a therapeutic intervention for patients with musculoskeletal disorders: a systematic review	BFR training is a safe intervention for musculoskeletal disorders with adverse events rarely reported.
Spranger et al., 2015 [31]	Blood flow restriction training and the exercise pressor reflex: a call for concern	BFR training raises concern in individuals with cardiovascular disease due to unknown effects of exercise pressor reflex on cardiovascular health.
Cristina-Oliveira et al., 2020 [32]	Clinical safety of blood flow-restricted training? A comprehensive review of altered muscle metaboreflex in cardiovascular disease during ischemic exercise	Concerns exist for BFR training in individuals with cardiovascular risk such as hypertension, heart failure, and peripheral artery disease.
Nascimento et al., 2022 [33]	A useful blood flow restriction training risk stratification for exercise and rehabilitation	Blood flow restriction training has risks for individuals with cardiovascular disease, impaired blood coagulability, or other comorbidities.

In strengthening the upper extremities, high-load resistance training has been the gold standard, which focuses on mechanical tension that forces a high amount of stress on the muscle to achieve muscle growth. Muscles in the upper extremity are generally smaller and more fragile, such as the rotator cuff muscles, biceps brachii, brachialis, and triceps brachii. Because of this, exercises that focus on high mechanical tension can more easily lead to injury. BFR training typically focuses on low-load resistance, which reduces the amount of stress on the muscle yet yields similar results in regard to muscle growth when compared to the traditional high-load training in the upper extremities [6,7,17]. Although BFR training is more commonly used with low-load resistance training, its use in high-load resistance training has also been assessed. When performed with high-load resistance training, the BFR group had superior strength gains, whereas the BFR with a low-load group demonstrated improved force precision control [18].

When performing BFR training on the arm, three locations can be affected in proximity to the cuff, which includes distally, proximally, and contralaterally. Distal to the cuff includes the biceps and forearms, and research has shown that BFR training can be very effective [6,19]. Proximal to the cuff includes the shoulder, trunk, chest, and hips and has shown mixed findings in the literature, but some articles have shown it to be effective in increasing strength and hypertrophy potentially due to a systemic increase in hormones and neural activation [6,19]. Contralateral to the cuff is the limb that is not being exercised and is a phenomenon known as cross-education where the motor cortex enhances activation of the muscles on the opposite side. Cross-training operates through a combination of physiological and neuromuscular mechanisms, allowing muscle activation and adaptation. It is believed that cross-training is due to neural factors [20]. Studies have shown that when unilateral resistance is performed on one extremity, there are increases in electromyography (EMG) and motor discharge rates for the contralateral limb muscles [21,22]. Also, because BFR training induces systemic hormonal responses, this may contribute to muscle hypertrophy and strength gains in the contralateral limb [20].

An article by Hill et al. [17] assesses the difference between eccentric and concentric BFR. Eccentric contraction refers to muscle lengthening as tension increases, whereas concentric contraction is where the muscle shortens as tension rises. The study found that eccentric BFR increased muscle strength in the contralateral untrained arm [11]. In a study by Bowman et al. [14], it was found that BFR training is effective in strengthening the muscles distally, proximally, and contralaterally.

When performing BFR it has been theorized that there may be different outcomes if different sized cuffs were used but according to Laurentino et al. [19], both narrow and wide cuffs can be used effectively for increasing muscle size and strength suggesting that clinicians should choose a cuff based on patient comfort levels [19,23].

The most common practice of applying BFR is continuously throughout the entire exercise, including rest. However, studies have explored the effects of intermittent BFR, where the cuff is deflated during rest and reinflated during exercise [17]. Additionally, understanding the effects of varying pressure levels has been crucial for exercise regimens to be successfully completed, for instance, using high pressures (~14.2 kg) during intermittent BFR application versus low pressures (~3.6kg) for continuous BFR periods compared to the baseline of ~17.7 kg [15,17]. Another aspect of BFR is the different applications (intermittent or continuous) and their effect on hemodynamics. 

In the study conducted by Bradner et al. [15], it was discovered that light-load exercise with continuous low-pressure BFR was preferred over light-load exercise with intermittent high-pressure BFR due to the decrease in hemodynamic stress, but all hemodynamic variables of heart rate, blood pressure, and rate-pressure product returned to baseline once the trials ended [15,17]. However, intermittent high-pressure BFR yielded a much higher heart rate and blood pressure than continuous low-pressure BFR, which means that intermittent BFR would require an increase in myocardial work [15,17]. This means that by utilizing the continuous low-pressure BFR, there is a decrease in potentially acquiring a thrombus along with a reduction of complete occlusion of the artery. Therefore, it was discovered that by utilizing the lower pressure continuous BFR, there would be a decrease in hemodynamic response as well as improvement in the comfort of the patient during the exercise. This study shows potential for a population with cardiovascular complications, such as in the elderly community, to help facilitate strength training in a more comfortable and less strenuous method.

The study by Roehl et al. examined the effects of different levels of limb occlusion pressures (LOP) at 0%, 25%, 50%, and 75%, focusing on the deltoid, middle deltoid, posterior deltoid, infraspinatus, teres minor, and trapezius. Participants performed three different rotator cuff exercises, including external rotation, internal rotation, and scaption at low intensity (20% maximal strength), and muscle activation was measured using EMG. The study found that higher occlusion pressures lead to increased muscle activation peaking at 50% LOP for most muscles [24]. Internal rotation led to increased activation of the anterior deltoid and trapezius at 25% LOP and teres minor at 75% LOP. Scaption led to an increased activation of the infraspinatus and teres minor at 25%, trapezius at 50%, and posterior deltoid at 75% LOP [24]. Although there was greater muscle activation, higher occlusion pressures led to faster fatigue and increased discomfort past 50% LOP, with participants reaching failure the quickest at 75% LOP. Overall, it was concluded that the optimal range is around 50% LOP in order to optimize muscle activation while minimizing fatigue and discomfort [24]. These findings show that while higher occlusion pressures can enhance muscle activation, they also increase discomfort and can reduce the number of repetitions, suggesting that there needs to be a balance between these factors when utilizing BFR in the upper extremity.

Although the study conducted by Bradner et al. [15] discovered effects on the cardiovascular system, a study conducted by Curty et al. [18] did not yield similar findings. By conducting the study solely comparing eccentric exercise sessions with and without BFR, it was concluded that no significant changes occurred hemodynamically between the two groups [1]. Therefore, future research is necessary to establish a standardized protocol for BFR training, including comparisons between intermittent and continuous pressure as well as high versus low-pressure BFR. These studies would be crucial in examining how these different methods would affect hemodynamics and muscle strength.

BFR training has an impact on muscle damage and recovery. When it comes to muscle damage, some of the markers to evaluate are arm circumference, range of motion (difference between the extended and flexed elbow joint angle), and muscle soreness upon palpation using the visual analog scale [1]. According to the study, a high-load eccentric exercise with BFR caused reduced muscle damage in the biceps brachii compared to high-load training without BFR [1,10]. This could be due to four factors; first, an increase in calcium, second an accumulation of metabolites intramuscularly, third an increase in fiber, and fourth a decrease in neutrophils, which helps reduce muscle inflammation [1]. Therefore, edema can be ruled out as the cause of increased muscle thickness following high-load eccentric exercise with BFR because muscle damage does not occur with BFR [1,25]. However, the BFR group experienced an increase in soreness due to elevated levels of metabolic products and hormonal response. This, in turn, results in the enhancement of type 4 nociceptor and pain receptor fibers, potentially leading to the soreness that might occur due to resistance training with BFR [1].

Clinical applications

The use of BFR in rehabilitation and injury recovery is crucial for not only athletes aiming to return to their sport in a timely manner but also the general population who are aiming to maintain their strength. People with an injury, such as rotator cuff tendinopathy, face the challenge of keeping up their strength in order to continue participating in their day-to-day life or sport after recovery. For example, rotator cuff injuries are difficult to recover from due to the poor blood supply, complex anatomy, and need for care during rehabilitation. When it comes to strengthening the shoulder, high-load training is risky and can even worsen the injury; therefore, low-load training is a different approach that could be safer along with the usage of BFR to further strengthen the muscles. In a study conducted by Kara et al. [10], it was discovered that biceps brachii muscle thickness and shoulder internal rotation strength increased in the rotator cuff tendinopathy with the BFR group compared to the control group. It was further believed that an increase in internal rotation strength could be due to the strengthening of the proximal muscles in the shoulder and chest [19]. This discovery was also found in the study by Lambert et al. [16], where BFR resulted in an increase in shoulder muscle mass and endurance, which potentially could be explained by the activation of the shoulder and chest muscles such as the deltoid and the rotator cuff muscles [7]. Although an increase in strength of the biceps brachii muscle was found, no significant difference in the reduction of pain or improvement of shoulder function with rotator cuff tendinopathy could be made [19].

Low-weight BFR training has not only increased strength and muscle hypertrophy but has the ability to increase grip strength in the contralateral arm (non-BFR extremity) explained by potentially a systemic effect [6]. However, another study found that BFR assisted the distal muscles in rock climbers by increasing grip and arm endurance rather than grip and arm strength [26].

BFR training not only can be incorporated into everyday exercise for people with injuries but also in sports. An example of BFR training in sports is seen in the study by Lambert et al. [26], in division IA collegiate baseball pitchers. This study focused on the use of BFR proximally for the shoulder in collegiate baseball players and found that low-load resistance exercise with BFR increased lean mass and endurance in the shoulder while maintaining rotator cuff strength [26,27]. This finding suggests the benefits of low-load resistance exercise with BFR in athletes that use an overhead or throwing motion in their sport, such as baseball players, in order to help prevent shoulder injuries while also strengthening the muscles in the shoulder.

Future directions

BFR training has opened doors for patients who are unable to lift heavy weights such as those with osteoarthritis, rotator cuff tendinopathy/impingement, muscle strain, labral repairs, post arthroplasty of the shoulder or elbow, or upper extremity traumatic injuries [6]. The reason for this unique modality is the fact that BFR training allows for an increase in strength without the mechanical stress on the tissues or joints that would normally occur in training without BFR. By incorporating this form of therapy, a quicker return to postoperative strength can occur along with a decrease in muscle atrophy [6].

BFR training should be performed or taught by professionals to ensure the correct application of the technique [28]. Sessions should be given to familiarize participants and help minimize any adverse side effects. Some potential side effects include soreness, tingling, numbness, and bruising [29]. Reports have shown more serious side effects of rhabdomyolysis, syncope, pulmonary embolism, and deep vein thrombosis (DVT) [2,6,28,30]. BFR training is contraindicated for individuals with cardiovascular disease, including those with hypertension, heart failure, or peripheral artery disease due to higher risk of a cardiovascular event [31,32]. Additionally, patients with a history of DVT or thromboembolic conditions should also avoid BFR training due to higher risk of thrombosis [33]. Overall, these studies present valid reasons to incorporate BFR into athletes’ training in order to prevent injuries and improve performance.

## Conclusions

In conclusion, BFR training has shown many substantial benefits in enhancing muscle strength, size, and endurance in the upper extremities across a variety of populations. This technique is valuable for athletes wanting to improve their performance. Low-load BFR has demonstrated similar effects to high-load exercises while minimizing cardiovascular and hemodynamic stress and mechanical strain on the tissues and joints. BFR has a decreased risk of muscle damage, which can be especially useful for older populations who cannot perform high-load activities or those who are recovering from a sensitive injury. Future research is still necessary, specifically on the effects of BFR on upper extremity injuries as well as a standardized protocol such as continuous versus intermittent BFR, high vs low-pressure BFR, and types of exercises. Overall, BFR is an innovative approach to exercising that may be beneficial for a variety of populations, making it a versatile tool in clinical and athletic settings.
